# Correction: Immediate effects of visual feedback on the accuracy of foot landing adjustments in older people with diabetes mellitus: A cross-sectional study

**DOI:** 10.1371/journal.pone.0331116

**Published:** 2025-08-26

**Authors:** Suzanne Martin, Simon B. Taylor, Shabnam Pejhan, Blynn L. Shideler, Rajna Ogrin, Rezaul Begg

The fourth author’s name is spelled incorrectly. The correct name is: Blynn L. Shideler.
