# Supplementary material for: Immediate effects of visual feedback on the accuracy of foot landing adjustments in older people with diabetes mellitus: A cross-sectional study
Source: PLoS One. 2025 May 21;20(5):e0323569. doi: 10.1371/journal.pone.0323569 (PMC12094761; doi:10.1371/journal.pone.0323569)
Supplement: Attachment — Submitted filename: Plos One6.doc [file pone.0323569.s001.doc]

Reviewer report Plos One

*Immediate effects of visual feedback on the accuracy of foot landing adjustments in older people with diabetes mellitus*

**General comments**

Thank you for giving me the opportunity to review this manuscript. The scope of the study is relevant to the readers of PlosOne. The study is interesting, but the manuscript has flaws which must be addressed before it can be suitable for publication. My main concern is the lack of a specified aim and that the method section is hard to follow. Please find my specific comments below.

**Title**

I suggest adding the design of the study to the title

**Abstract**

Baseline differences between the groups can be deleted, it is obvious that the group with diabetes differed in that aspect from the other groups and from the younger group in the aspects of diabetes and age.

I acknowledge that there is a word limit in abstract, but I still recommend the authors to include conclusion in the abstract.

**Introduction**

Line 55: Ref 5 is used for the statement that around 30-50% of falls cause minor injuries, this is not correct, ref 5 concerns mortality rates after fracture.

Line 68-70: please add a reference.

Line 75-77: The sentence “Compared with young adults, older people increase one shorter step and spent more time in double support when responding to an obstacle and a long step length target”. This sentence is hard to understand, what do you want to say with the sentence? I had to read the reference to be able to understand.

Line 86-87: The sentence “Therefore, this study used a more developed visual feedback system to enhance the accuracy of foot displacement adjustments in the sagittal plane.” This should be moved to method.

**Aim**

Aim is lacking, there are only hypotheses. Please add an aim for the study.

**Materials and methods**

All information is given in a single paragraph, and all information is mixed. I strongly advice the authors to structure the materials and methods section. Start with stating the design of the study. Gather all information about setting in one paragraph, participants in one paragraph, procedure in one etc.

**Results**

Line 148-149: information that data collection was performed at the Biomechanis laboratory at Victoria University should be moved to methods (settings).

Line 147-148 and line 157-158: the same information.

Line: 165: Delete “Mean”.

**Discussion**

What seems to be an aim is stated in the first sentence. The first paragraph in the discussion preferably includes main findings.

Line 239-242: It is very difficult to understand what you mean with the sentence starting with “When visual feedback is received”. Please rephrase. Also, this information (presented in a way that is understandable) should be given before the sentence “”Furthermore, reduced errors during steps 2-10 in the group of older adult with diabetes compared with errors in Step 1 can be related to corticospinal involvement”.

Line 291 and line 301: the word “elderly” is considered as ageism and should be avoided. Please check the entire manuscript. [Don’t call me “old”: Avoiding ageism when writing about aging | National Institute on Aging](https://www.nia.nih.gov/research/blog/2023/12/dont-call-me-old-avoiding-ageism-when-writing-about-aging)

**Conclusion**

Conclusion should relate to the aim. Please check the conclusion after you have added an aim. Also move the sentence about implications for future research to the end of the discussion.

**Tables and figures**

Table 1: add “gait” to velocity.

**References**

Ref 5 is not adequat.
